# Quality of Care for Patients with Acute Myocardial Infarction (AMI) in Pakistan: A Retrospective Study

**DOI:** 10.3390/ijerph16203890

**Published:** 2019-10-14

**Authors:** Shazia Rehman, Xi Li, Chao Wang, Muhammad Ikram, Erum Rehman, Meina Liu

**Affiliations:** 1 Department of Biostatistics, School of Public Health, Harbin Medical University, No.157 Baojian Road, Harbin 150081, China; s.rehmaan@outlook.com (S.R.); lixi_094@163.com (X.L.); wangchao_ln1219@163.com (C.W.); 2 College of Economics and management, Nanjing University of Aeronautics and Astronautics, 29 Jiangsu Avenue, Nanjing 211106, China; mikram@nuaa.edu.cn; 3 School of Mathematics and Statistics, Southwest University of Finance and Economics, Chengdu 610074, China; 117020208901@smail.swufe.edu.cn

**Keywords:** acute myocardial infarction, quality of care, in-hospital outcomes, compliance measurement, composite score

## Abstract

A wide variation exists in the practice patterns of acute myocardial infarction (AMI) care worldwide, leading to differences in clinical outcomes. This study aims to evaluate the quality of process care and its impact on in-hospital outcomes among AMI patients in Pakistan, as no such study has been conducted in Pakistan thus far based upon recommended guidelines. We investigated a sample of 2663 AMI patients across 11 territory hospitals in Punjab province of Lahore, Faisalabad, Multan, Rawalpindi, and Islamabad from January 1, 2016 to December 31, 2017, with an in-hospital mortality rate of 8.6%. We calculated compliance rates of quality indicators (QIs) for all eligible patients. The association between process care and in-hospital outcome was assessed using hierarchical generalized linear model that adjusted for patient and hospital characteristics. In addition, we examined the effect of patient composite scores on clinical outcomes. Aspirin (73.08%) and clopidogrel (67.86%) indicated relatively better conformance than other QIs. The percutaneous coronary intervention also showed significantly low adherence. All QIs showed no significant association with in-hospital mortality. In contrast, 4 out of 8 QIs were observed positively correlated with in-hospital length of stay (LOS). The overall patient composite score was found to be statistically significant with in-hospital LOS. The assessment of quality of care showed low adherence to clinical care recommendations, and increased adherence was associated with longer in-hospital LOS among AMI patients. Evaluation of valid QIs for AMI treatment and their impact on in-hospital outcomes is an important tool for improving health care delivery in the overall AMI population in Pakistan. Low adherence to performance measures strongly compel to focus on guideline-based tools for AMI in Pakistan.

## 1. Introduction

Despite attempts by professional societies to standardize acute myocardial infarction (AMI) care through the guidelines, substantial variation exists in the practice patterns for the management of AMI in hospitals around the world. In the era of accountability in the therapeutic field, evaluation of quality of care plays a vital role and has turned into an imperative tool for health specialists and experts [1]. However, assessing the quality of care in patients admitted for acute myocardial infarction (AMI) is challenging because it is a complex notion that is based on a wide broad variety of factors not restricted to positive clinical results only [2]. Therefore, evaluation of the quality of care by quality indicators (QIs) has become a standard, global practice that captures the detailed outcome of treatment programs accurately [3,4,5,6]. Several countries and territories have introduced a number of QIs to evaluate and assess the quality of health care in AMI patients [7,8,9,10,11,12]. In a few western countries, the implementation of QIs for AMI patients has delivered significant outcomes and has likewise shown their capacity to identify fluctuations in care across hospitals [2,3,6,13].

AMI is among the leading causes of death and disability worldwide. It has a strong significance in accomplishing rapid revascularization due to on-time diagnosis and treatment which largely affects survival [14]. Accurate burden assessment of patients with AMI in low- and middle-income countries is mostly unavailable; however, crude estimates indicate that the burden of AMI has been steadily increasing over the last decades in low- and middle-income countries [15]. Cardiovascular risk factors for ischemic heart disease and AMI are on the rise in Pakistan and a leading cause of morbidity and mortality [16]. However, Pakistan is spending less than 1% of the gross domestic product (GDP) on public health for the past few decades, while the World Health Organization (WHO) benchmark of health expenditure is at least 6% of the GDP [17]. 

Although individual QIs are useful to measure the outcomes for improvement activities, questions remain concerning how to use the available data to assess and quantify progress made in quality improvement. Composite measures aggregate individual QIs into a summary of performance across multiple dimensions of care, which allows an overall representation of healthcare quality provided and provides a more understandable format. To date, there has been no investigation done in context of Pakistan for assessing the quality of AMI care based upon the recommended guidelines or the association between the QIs and in-hospital outcomes. Previous studies [18] have emphasized the association between process of care measures and in-hospital outcome, but have been limited ascertainment of in-hospital events, which can be substantially biased by in-hospital length of stay. Thus, the degree to which process measure performance conveys meaningful information about in-hospital mortality remains unclear. To address the above-mentioned needs, we aimed to estimate the compliance rate of QIs, and examine the association between quality indicators care and in-hospital outcomes among ST Segment Elevation Myocardial Infarction (STEMI) and Non-ST Segment Elevation Myocardial Infarction (NSTEMI) patients, including individual QIs and patient composite score. Identifying valid QIs for AMI treatment and their impact on in-hospital outcomes, is an important tool for improving the quality of health care.

To the best of our insight, this study is the first of its kind done in the Pakistani population to assess the relationship between quality care and in-hospital outcomes among AMI patients. The primary quality of our investigation is that it provides the most recent statistics for evaluating quality of care delivery for patients with AMI in Pakistan. Also, it provides an estimation of composite score from the patient dimension using quality indicators.

## 2. Methods

### 2.1. Data Source and Study Population

A retrospective study plan has been carried out in 11 Coronary Care Units (CCU), in tertiary of Punjab, Pakistan. Information was derived from administrative hospital discharge data of AMI patients from 1 January 2016 to 31 December 2017 by three trained bio-statisticians. This study chooses the big cities of Punjab, such as Faisalabad, Lahore, Multan, Rawalpindi, and Islamabad, to measure the quality of care and its impact on in-hospital outcomes among AMI patients who have a strong economic background. The details of geographical, racial and economic factors of Punjab cities are available at the Pakistan Bureau of Statistics [19]. The study population consisted of patients who presented to emergency room within 24 h of the onset of an ischemic syndrome, and the ultimate primary diagnosis was AMI labeled as either STEMI or NSTEMI. Variables include patient demographics, clinical presentation, medical history, cardiovascular risk factors, co-morbidities, timings of care conveyance; treatments advised as well as major contraindications related to treatments, post-hospital discharge therapy, and in-hospital patient outcomes.

For hospitals with more prominent than 300 AMI cases, we excluded those that had no patient’s outcome(death/survival). Thus, we were left with only 300 cases. For hospitals fewer than 300 AMI cases, all were chosen. Patients were excluded in the final analysis on the off chance that they were hospitalized for more than 90 days, in the event that they were transferred from another acute care facility, in the case they had stayed < 1 day and discharged alive, and those with had lost information. After avoidance, a total of 2663 AMI cases—1454 STEMI and 1209 NSTEMI, were cleared out for final investigations. Ethical approval for the study was obtained from Ethics Review Committee of all hospital regulatory body Ministry of health Pakistan approval reference (KIIT 2019/PK 2019-25- MS 65). 

### 2.2. Quality Indicators

Quality of care for AMI patients was investigated using 13 QIs at the primary and secondary levels. The relationship between adherence to these indicators and in-hospital outcomes was evaluated. The indicators of quality care measures included were the in-hospital advise of aspirin (QI1), beta blockers (QI2), clopidogrel (QI3), thrombolytic (QI4), electrocardiograph (ECG) (QI5), left ventricular function(LVF) assessment (QI6), coronary angiography (QI7), primary percutaneous coronary intervention(PCI) (QI8) and at secondary level, use of aspirin (QI9), beta-blocker (QI10), Angiotensin-Converting Enzyme (ACE) Inhibitor (QI11), Statin (QI12), Clopidogrel (QI13) at post-discharge. These measures are proposed by the Chinese health care system based upon the Modified Delphi process [20]. Unlike the indicators proposed by the American College of Cardiology and the American Heart Association (ACC/AHA), which consisted of only quality of care measures [21], The present investigation included additional structural and outcome indicators, based on the theoretical structure of Donabedian medical quality system [20,21,22,23]. Due to many differences between Pakistan and other developed countries when comparing health-care systems and how diseases are treated, we focused on using Chinese quality indicators for AMI care as both are developing countries. In-hospital mortality and length of stay (LOS) were considered as outcome measures.

### 2.3. Statistical Analysis

Baseline demographic characteristics of patients and co-morbidities for the selected population were summed up in the form of frequencies and percentages (Table 1 and Table 2). In addition, for each baseline group, in-hospital mortality rate and LOS were also calculated as the outcome might also be influenced by patient’s characteristics. We conducted a univariate analysis to examine the effects of patient characteristics and comorbidities on patient outcomes. Each QI was an independent variable separately and in-hospital outcome (mortality/LOS) was the dependent variable. The adherence rate was calculated for each QI as the sum of patients receiving care (numerator) divided by the number of patients meeting eligibility criteria (denominator) [20]. We calculated the patient composite score using the sum of numerator divided by the sum of denominator of all QIs. With patient-level and hospital-level risk factors as fixed effects and a random intercept for hospitals, unadjusted and adjusted hierarchical generalized linear model (HGLM) were used to investigate the impact of receiving treatment (usage of a single indicator/patient quality of care composite score) on in-hospital outcomes. For each QI, patients meeting the eligibility criteria were included in the analysis. The composite score was added as a continuous predictor variable, and regression coefficients were reported per 10% increment in composite score. All significant (*p* < 0.05) risks factors (listed in Table 1 and Table 2) in univariate analysis for patient characteristics were co-variables in the HGLM. Analysis of the abstracted data was performed using SAS version 9.3 (SAS Institute Inc., Cary, NC, USA).

## 3. Results

### 3.1. Clinical Characteristics

**Figure 1:**
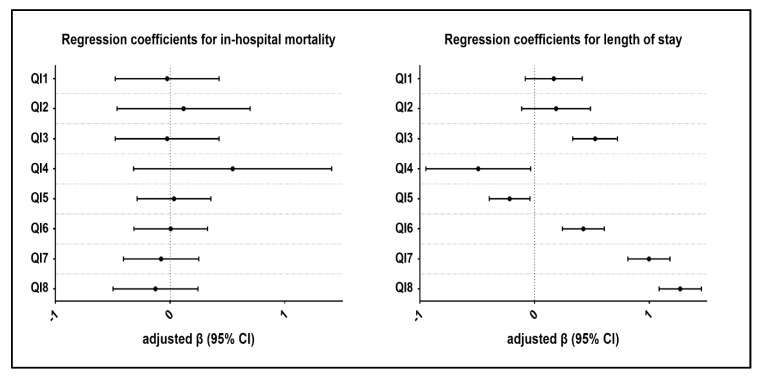
Regression coefficients estimation for in-house mortality and length of stay.

Table 1 indicates the baseline and clinical attributes of admissions for AMI. Among 2663 patients, 1859 (69.81%) of the admissions were male patients. In-hospital mortality rate accounted approximately same for both males and females as 8.55% and 8.58%, respectively. An aggregate of 1197 patients was > 70 years old with a higher death rate of 9.11%. 2314 patients were reported as obese, with a mortality rate of 8.69%. Only 38% had health insurance. 56.37% were smokers and demonstrated a slightly higher rate of in-hospital mortality, i.e. 8.86% than those without smoking, i.e. 8.18%. Approximately, half of them had a family history of ischemic heart disease (42.32%). Among 2663 patients, 1052 had a background marked by hypertension and 909 had a history of diabetes mellitus. A number of STEMI admissions was more prominent than NSTEMI. However, the mortality rate was reported higher for NSTEMI admission. Similarly, patients admitted in generalized hospitals were significantly higher than the specialized hospital type, though specialized hospitals showed 9.96% mortality rate higher than the generalized one. The in-hospital mortality rate was 8.6% for our data sample. The mean and standard deviation of LOS was observed significantly associated with patient characteristics including age (for all group), obese (yes/no), insurance (Govt/private), prior myocardial infarction/coronary artery disease (MI/CAD: had/no), history of hypertension (had/no), type of MI (STEMI/NSTEMI) and hospital type (specialized/generalized). Table 2 illustrates the detailed statistics of comorbidities associated with in-hospital mortality and length of stay, also presented in Figure 1. Our findings show that patients with rheumatic heart disease and cerebrovascular disease at time of admission were observed significantly associated with in-hospital mortality. On the other hand, patients conceded with cardiogenic shock, higher level of lipids and thyroid as a comorbid factor were more likely to correlate with an increase in LOS.

### 3.2. Compliance Measurements

At a primary level of care,1132 of 1549 patients (73.08%) with AMI furnished with aspirin within three hours of hospital arrival. 785 patients were eligible for receiving beta-blockers, only 306 (38.98%) actually received it. An aggregate of 1634 of 2408 patients (67.86%) was facilitated with clopidogrel. Thrombolytic appeared with the lowest compliance rate of 7.52%. ECG, left ventricular ejection fraction (LVEF) assessment, and coronary angiography were recommended to every chosen patient however just 1664 (62.49%), 1672 (62.79%), and 1767 (66.35%) patients were given the exhorted therapy, individually. Of 2366 patients who were considered as eligible for percutaneous coronary intervention (PCI) treatment, only 884 (37.36%) acquired it. At the secondary dimension of consideration, aspirin appeared with a compliance rate of 47.97%, beta-blocker with 22.29%, clopidogrel with 45.35%, statin with 36.39%, and ACE inhibitor with 38.02%. Among all indicators (at the primary and secondary level of care), the most noteworthy compliance rate was of aspirin whereas, thrombolytic showed the least rate (Table 3).

Aspirin and clopidogrel being pharmacological indicators showed better conformance at hospital level of care whereas in the case of thrombolytic poorer adherence was observed. For PCI, as an invasive procedure, there was no significant conformance observed across hospitals. At secondary level of prevention, hospital adherence was slightly on the rise but yet not satisfactory (Table 3). The median (Interquartile range) composite score for patients was 0.50 (0.38–0.63).

### 3.3. Association between Process Indicators and Outcomes

Table 4 summarizes the results of adjusted and unadjusted regression coefficients with 95% confidence interval for the association between process measures and patient outcome at discharge. 3 out of 8 QIs showed a negative association on in-hospital mortality, although insignificant, which indicates that patients who received aspirin, coronary angiography, and PCI during their hospital stay were at lower risk of dying in the hospital. After adjustment for confounding factors, the results remain unchanged, and no significant differences were observed. In contrast, 4 out of 8 QIs showed strong positive and significant association regarding LOS, which suggested that AMI patients who received clopidogrel, LVEF assessment, coronary angiography, and PCI during their hospital stay were more likely to be associated with an increased LOS. Overall PCI, turned out to be the largest risk factor on account of increased LOS whereas aspirin, beta-blockers and thrombolytic were not considered as predictive indicators.

Table 5 summarizes the impact of the patient composite scores on in−hospital mortality. Overall, the patient composite scores were observed insignificantly associated with in−hospital mortality (*p* > 0.05), although only one measure, i.e., STEMI vs. NSTEMI gives a statistically significant relationship at *p* = 0.05 level. However, at a more aggregate degree, overall composite score (per 10%) was insignificantly but positively associated with in−hospital deaths. The factor with the greatest weight affecting in−hospital mortality is rheumatic heart disease, although found insignificant. On the contrary, the effect of patient composite score on LOS gives a little different result. The effect of patient composite score on LOS for all age groups was found significantly and positively associated at *p* = 0.05 level of significance.

Interestingly, the effect of patient composite score on length of stay for all age groups was found significantly and positively associated at *p* = 0.05 level of significance. Also, STEMI vs. NSTEMI and thyroid patients ended up being statistically significant, with a positive association towards length of stay. More precisely, overall composite score (per10%) was observed statistically significant giving an indication of meaningful addition to our model (Table 6). The median (Interquartile range) composite score for patients was 0.50 (0.38–0.63). As can be seen from these two tables that STEMI vs. NSTEMI is the common factor of patient composite score which is associated with in−hospital mortality and LOS. The overall low quality of patient care was observed based on composite score.

## 4. Discussion

This study provides the most recent statistics for evaluating quality of care delivery for patients with acute MI in Pakistan. A well−prepared checklist was administered in order to get the characteristics of AMI patients. Further, this study has observed overall a low compliance rate, which is explicitly lower than the international standards among AMI patients (STEMI and NSTEMI). No significant association was observed between QIs and in−hospital mortality. 4 out of 8 quality indicators showed strong positive and significant association in terms of LOS. Likewise, patient composite score was also observed significantly associated with LOS. Composite performance measures are often attached to regulatory mechanisms whereby hospitals are rewarded or punished according to the outcome of the composite indicator. The use and publication of composite performance measures can generate both positive and negative behavioral responses [24].

Except for aspirin, other indicators all demonstrated poor compliance rates. This finding is inconsistent with a study from China that reported weak conformance for a few indicators [25]. The possible reasons for low compliance rate could be the lack of staff and resources at governmental hospitals. Lack of AMI quality improvement programs at hospital levels may be another reason for poor performance in AMI care management. Studies have suggested that AMI patients face significant delays before adequate treatment is available to them. Framework delays and access to reperfusion are distinguished as drivers of destitute compliance rate and remained significant challenges inside the AMI framework of care within the hospitals of Pakistan [15,26]. Execution in quality indicators measurement can, subsequently, be progressed by simply expanding adherence to these guidelines. However, the results indicate that serious attention is required to improve the steps of quality of care over 11 hospitals.

Every single indicator must be characterized with the end goal of assessment. Few indicators are time confined, while others such as coronary angiography and PCI are free from time restriction [11,27]. Both procedures cost high and most of the eligible patients can’t manage the cost of it. In any case, for a clear reason, it is still recommended as procedure of care indicator. The compliance rate should way to deal with 100% when every single qualified patient gets exhorted medication or therapy.

Nonetheless, because occasionally contraindications to advised therapy are not constantly enrolled, the recommended benchmark esteems underneath 100% [28]. It likewise indicates that there is still opportunity to get better notwithstanding the absence of consistency with guidelines. We found that in univariate analysis the effect of patient’s characteristics on LOS is more significant than in−hospital mortality. Also, in multivariate analyses adherence to QIs was found significantly correlated with LOS. Postoperative complications or comorbidities associated at time of admissions may be a reason for increased LOS.

Another finding of this study is the concept of a wide scope of comorbidities associated with the time of admission and their impact on clinical outcomes. The measurement of comorbidities remains an area of concern for scientists and clinicians [29]. Studies have shown that prolonged hospitalization, decreased quality of life, and increased health−care costs can be affected by comorbidities associated at the time of admissions for patients with AMI [30,31,32]. The main and foremost limitation of our study was inaccessibility of long−term outcome of the selected sampled population. As a result of no subsequent system in Pakistan, it was not in any way conceivable to induce data of these patients after discharge. Therefore, in comparison to others, this study only comprises of patient data during their hospital stay. Another impediment of this study was precision in numerators and denominators.

We believe these results give a credible source of valid information when assessing and improving the quality of medical care. Moreover, this source of information will allow an easier comparison, both within and between countries; this, in turn, leads to more accurate and trustworthy reporting to drive further assessment and improvement in quality of care and outcomes. Clinical indicators function as the spine for assessing the quality of care provided across the full spectrum of domains of patient care [33]. Therefore, monitoring the health care quality framework, these clinical measures assume an essential job. To ensure their unwavering quality and legitimacy, they should be developed and implemented with transparency.

The therapeutic records of patients in Pakistani hospitals are intended for administrative instead of evaluation purposes, which may have resulted in some evidence being missed or inaccurate. A few patients denied a recommended therapy or left against medical advice on account of their poor budgetary circumstances, so the numerator could be thought little off. Some data about contraindications may be erroneously recorded, causing an overestimation of the denominator. Besides, uncertain affiliations of value measures on healthcare procedures and mortality could likewise be clarified by non−medical care−related variables, e.g., financial status and local healthcare organizations that would influence the health outcomes after discharge. These variables were out of the control of the hospital and will exclusively affect the mortality proportion. Moreover, quality of care measurement depends on the detectability of information gathered in medicinal records and may not precisely reflect care conveyance.

## 5. Conclusions

This study in a resource−limited setting, showed some disparities between guidelines and clinical practice. Low adherence to performance measures strongly compel to focus on guideline−based tools for AMI in Pakistan. Taking everything into account, our analyses of the quality of care indicators for AMI patients reveal less satisfactory outcomes. We prescribe a more grounded spotlight on Pakistani national health services regulation and coordination in AMI care and the foundation of national benchmarks for AMI−explicit consideration thrombolytic and PCI related quality indicators to encourage the nature of consideration for AMI patients in Pakistan. Further investigations should be led in other provinces of Pakistan to distinguish, evaluate, and improve the AMI care. Besides, we recommend that clinicians and health care providers use both quality indicators and outcome measures to optimally assess overall clinic quality of care for AMI. The difference in treatment quality between hospitals shows that there is room for improvement in hospitals with poor quality, and it can also help hospitals to make targeted improvements.

## Figures and Tables

**Table 1:** Univariate relationship between patient characteristics and outcome measures.

Patient Characteristics	Frequency (%)	In-Hospital Mortality		LOS (day)	
		N (%)	*p* Value	mean ± SD	*p* Value
Age					
~50	316 (11.87)	26 (8.23)	0.8170	6.07 ± 3.05	<0.0001
50–60	414 (15.55)	32 (7.73)		7.27 ± 2.50	
60–70	736 (27.64)	61 (8.29)		7.64 ± 2.29	
70~	1197 (44.95)	109 (9.11)		7.41 ± 2.50	
Sex					
Male	1859 (69.81)	159 (8.55)	0.9803	7.33 ± 2.55	0.6370
Female	804 (30.19)	69 (8.58)		7.29 ± 2.59	
Obese					
No	2314 (86.89)	201 (8.69)	0.5544	7.37 ± 2.53	0.0045
Yes	349 (13.11)	27 (7.74)		6.96 ± 2.69	
Insurance					
Government	673 (25.27)	60 (8.92)	0.9177	7.16 ± 2.77	0.0040
Private	341 (12.81)	28 (8.21)		7.49 ± 2.78	
Uninsured	1649 (61.92)	140 (8.49)		7.70 ± 2.46	
Smoking					
No	1162 (43.63)	95 (8.18)	0.5308	7.32 ± 2.57	0.2555
Yes	1501 (56.37)	133 (8.86)		7.31 ± 2.55	
Family history of IHD *					
No	1536 (57.68)	132 (8.59)	0.9451	7.24 ± 2.58	0.1109
Yes	1127 (42.32)	96 (8.52)		7.42 ± 2.53	
Prior MI/CAD					
No	1693 (63.57)	153 (9.04)	0.2467	7.23 ± 2.58	0.0214
Yes	970 (36.43)	75 (7.73)		7.46 ± 2.58	
History Of DM					
No	1754 (65.87)	151 (8.61)	0.9039	7.26 ± 2.62	0.2349
Yes	909 (34.13)	77 (8.47)		7.41 ± 2.42	
History of Hypertension					
No	1611 (60.50)	130 (8.07)	0.2612	7.20 ± 2.40	0.0151
Yes	1052 (39.50)	98 (9.32)		7.48 ± 2.48	
MI					
NSTEMI	1209 (45.40)	124 (10.26)	0.0044	6.09 ± 2.65	<0.0001
STEMI	1454 (54.60)	104 (7.15)		8.33 ± 1.96	
Hospital type					
Specialized	1034 (38.83)	103 (9.96)	0.0397	7.08 ± 2.64	<0.0001
Generalized	1629 (61.17)	125 (7.67)		7.46 ± 2.49	

* There is 1 missing value. Abbreviation: LOS, length of stay; SD, standard deviation; MI, Myocardial Infarction; IHD, Ischemic Heart Disease; DM, Diabetes Mellitus; CAD, Coronary Artery Disease; STEMI, ST-elevation myocardial infarction; NSTEMI, Non-ST-elevation myocardial infarction.

**Table 2:** Univariate analysis of comorbidities with in-hospital mortality and length of stay.

Comorbidities	Frequency (%)	In-Hospital Mortality		LOS (day)	
		N (%)	*p* Value	Mean ± SD	*p* Value
Rheumatic heart disease					
no	2655 (99.70)	226 (8.51)	0.0002	7.32 ± 2.56	0.8367
yes	8 (0.30)	2 (25.00)		6.88 ± 3.18	
Heart Failure					
no	2615 (98.20)	222 (8.49)	0.3251	7.31 ± 2.56	0.9827
yes	48 (1.80)	6 (12.50)		7.35 ± 2.37	
Cardiogenic Shock					
no	2392 (89.82)	204 (8.53)	0.8550	7.27 ± 2.57	0.0137
yes	271 (10.18)	24 (8.86)		7.68 ± 2.43	
Hypertension					
no	1998 (75.03)	167 (8.36)	0.5155	7.30 ± 2.57	0.8212
yes	665 (24.97)	61 (9.17)		7.35 ± 2.53	
Cerebrovascular disease					
no	2253 (84.60)	205 (9.10)	0.0022	7.29 ± 2.60	0.5060
yes	410 (15.40)	23 (5.61)		7.43 ± 2.32	
Gastrointestinal disease					
no	2620 (98.39)	226 (8.63)	0.3555	7.32 ± 2.56	0.5060
yes	43 (1.61)	2 (4.65)		7.05 ± 2.54	
Type-I DM					
no	2307 (86.63)	200 (8.67)	0.6138	7.29 ± 2.60	0.5357
yes	356 (13.37)	28 (7.87)		7.48 ± 2.26	
Type-II DM					
no	2322 (87.19)	197 (8.48)	0.7084	7.33 ± 2.56	0.4519
yes	341 (12.81)	31 (9.09)		7.23 ± 2.52	
Renal failure					
no	2451 (92.04)	206 (8.40)	0.3247	7.34 ± 2.54	0.3972
yes	212 (7.96)	22 (10.38)		7.00 ± 2.71	
Dysrhythmia					
no	2485 (93.32)	216 (8.69)	0.3689	7.29 ± 2.57	0.1766
yes	178 (6.68)	12 (6.74)		7.66 ± 2.69	
Peripheral Vascular Disease					
no	2389 (89.71)	201 (8.41)	0.4196	7.30 ± 2.58	0.9493
yes	274 (10.29)	27 (9.85)		7.41 ± 2.32	
COPD					
no	2525 (94.82)	215 (8.51)	0.7113	7.30 ± 2.56	0.1561
yes	138 (5.18)	13 (9.42)		7.61 ± 2.43	
Liver disease					
no	2615 (98.20)	224 (8.57)	0.9545	7.31 ± 2.56	0.4408
yes	48 (1.80)	4 (8.33)		7.60 ± 2.44	
Hypercholesterolemia					
no	1614 (60.61)	140 (8.67)	0.7972	7.22 ± 2.59	0.0499
yes	1049	88 (8.39)		7.46 ± 2.50	
Thyroid Disorder					
no	1950	175 (8.97)	0.2082	7.19 ± 2.62	<0.0001
yes	713	53 (7.43)		7.65 ± 2.34	

Abbreviation: LOS, length of stay; SD, standard deviation; COPD, chronic obstructive pulmonary disease; DM, diabetes mellitus.

**Table 3:** Adherence to performance measures.

Quality Indicators (QI)	Eligible Patients, No. (%) ^a^	Overall Adherence, %	Hospital Variation, Range (%)
QI1: Aspirin prescribed within 3h of hospital arrival	1549 (58.17)	73.08	62.11–85.81
QI2: Beta-blockers within 12h of arrival	785 (23.69)	38.98	32.89–45.45
QI3: Clopidogrel within 12h of arrival	2408 (90.42)	67.86	62.68–73.27
QI4: Thrombolytics received within 30 min of hospital arrival	971 (36.46)	7.52	3.57–11.43
QI5: ECG within 10 min of hospital arrival	2663 (100.00)	62.49	57.74–71.84
QI6: Left ventricular function assessment	2663 (100.00)	62.79	56.33–67.53
QI7: Coronary angiography performed during hospital stay	2663 (100.00)	66.35	60.85–71.78
QI8: Primary PCI	2366 (88.85)	37.36	29.52–43.59
Prescription filled post-discharge			
QI9: Aspirin	1549 (58.17)	47.97	38.51–61.29
QI10: Beta-blocker	785 (29.48)	22.29	10.94–34.21
QI11: Clopidogrel	2408 (90.42)	45.35	35.36–53.59
QI12: Statin	2020 (75.85)	36.39	30.11–41.76
QI13: ACE inhibitor	2567 (96.40)	38.02	29.70–45.54
Outcome indicators			
In-hospital mortality	2663 (100.00)	8.56	5.56–12.50

Abbreviation: PCI, percutaneous coronary intervention. ^a^ Eligible patients are those with definite indications but no documented contraindications.

**Table 4:** Association of process measures with clinical outcomes among acute myocardial infarction patients.

Quality Indicators^*^	In−Hospital Mortality		Length of Stay	
	Adjusted β (*p* Value)	Unadjusted β (*p* Value)	Adjusted β (*p* Value)	Unadjusted β (*p* Value)
QI1	−0.0258 (0.9014)	−0.0382 (0.8540)	0.1668 (0.1880)	0.2930 (0.0340)
QI2	0.1174 (0.6616)	0.1079 (0.6856)	0.1873 (0.2207)	0.2337 (0.1456)
QI3	0.0238 (0.8831)	0.0156 (0.9229)	0.5278 (<0.0001)	0.7994 (<0.0001)
QI4	0.5451 (0.1898)	0.6093 (0.1457)	−0.4913 (0.0351)	−0.4994 (0.0328)
QI5	0.0342 (0.8177)	0.0525 (0.7229)	−0.2179 (0.0159)	−0.2913 (0.0043)
QI6	0.0048 (0.9742)	−0.0636 (0.6647)	0.4251 (<0.0001)	0.8413 (<0.0001)
QI7	−0.0786 (0.6057)	−0.1535 (0.3110)	0.9974 (<0.0001)	1.5171 (<0.0001)
QI8	−0.1276 (0.4603)	−0.2174 (0.2102)	1.2699 (<0.0001)	1.7515 (<0.0001)

* The definition of quality indicators (QIs) please refer to Table 3. All significant (*p* < 0.10) risks factors (listed in Table 1) in univariate analysis for patient characters were included in the hierarchical generalized linear model.

**Table 5:** Effect of patient composite scores on in−hospital mortality.

Factors	Regression Coefficient, 95%CI	*p* Value
STEMI vs. NSTEMI	1.451 (1.026, 2.053)	0.0379
Generalized vs. Specialized Hospital	0.877 (0.613, 1.254)	0.4270
Cerebra disease	0.620 (0.355, 1.082)	0.0849
Rheumatic Heart Disease (RHD)	2.346 (0.070, 78.121)	0.4952
Composite score (per 10%)	1.003 (0.937, 1.073)	0.9284

The composite score was added as a continuous predictor variable, and regression coefficients were reported per 10% increment in composite score. Abbreviation: STEMI, ST-elevation myocardial infarction; NSTEMI, Non-ST-elevation myocardial infarction.

**Table 6:** Effect of patient composite scores on length of stay.

Factors	Regression Coefficient, 95%CI	*p* Value
Age		
50–60 vs. ~50	0.481 (0.132, 0.829)	0.0069
60–70 vs. ~50	0.751 (0.432, 1.071)	<0.0001
70~ vs. ~50	0.587 (0.282, 0.891)	0.0002
Insurance		
Government vs. Uninsured	0.147 (−0.059, 0.354)	0.1623
Private vs. Uninsured	0.202 (−0.069, 0.474)	0.1443
Prior MI/CAD	0.021 (−0.210, 0.251)	0.8595
History of Hypertension	0.137 (−0.091, 0.365)	0.2401
STEMI vs. NSTEMI	2.049 (1.871, 2.227)	<0.0001
Generalized vs. Specialized hospital	0.168 (−0.164, 0.499)	0.3213
thyroid	0.257 (0.058, 0.455)	0.011
lipids	0.179 (−0.001, 0.359)	0.051
Composite score (per 10%)	0.258 (0.217, 0.299)	<0.0001

The composite score was added as a continuous predictor variable, and regression coefficients were reported per 10% increment in composite score. Abbreviation: MI, Myocardial Infarction; CAD, Coronary Artery Disease; STEMI, ST-elevation myocardial infarction; NSTEMI, Non-ST-elevation myocardial infarction.
